# NIR Luminescent Oxygen-Sensing Nanoparticles for Continuous Glucose and Lactate Monitoring

**DOI:** 10.3390/bios13010141

**Published:** 2023-01-14

**Authors:** Ananthakrishnan Soundaram Jeevarathinam, Waqas Saleem, Nya Martin, Connie Hu, Michael J. McShane

**Affiliations:** 1 Department of Biomedical Engineering, Texas A&M University, College Station, TX 77843, USA; 2 Materials Science and Engineering, Texas A&M University, College Station, TX 77845, USA

**Keywords:** Ethyl cellulose, nanoparticles, near infrared, oxygen sensor, glucose sensor, lactate sensor, NIMs

## Abstract

A highly sensitive, biocompatible, and scalable phosphorescent oxygen sensor formulation is designed and evaluated for use in continuous metabolite sensors for biological systems. Ethyl cellulose (EC) and polystyrene (PS) nanoparticles (NPs) stabilized with Pluronic F68 (PF 68), Polydimethylsiloxane-*b*-polyethyleneglycol methyl ether (PDMS-PEG), sodium dodecylsulfate (SDS), and cetyltimethylammonium bromide (CTAB) were prepared and studied. The resulting NPs with eight different surfactant–polymer matrix combinations were evaluated for physical properties, oxygen sensitivity, effect of changes in dispersion matrix, and cytotoxicity. The EC NPs exhibited a narrower size distribution and 40% higher sensitivity than PS, with Stern–Volmer constants (*K_sv_*) 0.041–0.052 µM^−1^ for EC, compared to 0.029–0.034 µM^−1^ for PS. Notably, ethyl cellulose NPs protected with PF68 were selected as the preferred formulation, as they were not cytotoxic towards 3T3 fibroblasts and exhibited a wide phosphorescence lifetime response of >211.1 µs over 258–0 µM and ~100 µs over 2.58–0 µM oxygen, with a limit of detection (LoD) of oxygen in aqueous phase of 0.0016 µM. The EC-PF68 NPs were then efficiently encapsulated in alginate microparticles along with glucose oxidase (GOx) and catalase (CAT) to form phosphorescent nanoparticles-in-microparticle (NIMs) glucose sensing microdomains. The fabricated glucose sensors showed a sensitivity of 0.40 µs dL mg^−1^ with a dynamic phosphorescence lifetime range of 46.6–197.1 µs over 0–150 mg dL^−1^ glucose, with a glucose LoD of 18.3 mg dL^−1^ and maximum distinguishable concentration of 111.1 mg dL^−1^. Similarly, lactate sensors were prepared with NIMs microdomains containing lactate oxidase (LOx) and found to have a detection range of 0–14 mg dL^−1^ with LoD of 1.8 mg dL^−1^ and maximum concentration of 13.7 mg dL^−1^ with lactate sensitivity of 10.7 µs dL mg^−1^. Owing to its versatility, the proposed NIMs-based design can be extended to a wide range of metabolites and different oxygen-sensing dyes with different excitation wavelengths based on specific application.

## 1. Introduction

Precise and reliable methods for the measurement of dissolved oxygen are important in the design of biosensors tailored for different life science applications, including in vivo metabolite monitoring, anaerobe culture, and bioprocess monitoring [1,2,3]. Beyond biomedicine, dissolved oxygen probes are also important for corrosion protection, semiconductor industry, water quality monitoring, and surface treatment [4,5,6,7,8]. Further, due to the ubiquitous nature of oxygen and its participation in many reactions of biological relevance, oxygen measurements are also often used to indirectly determine concentrations of other species (e.g., oxidation substrates of enzymatic reactions) [9].

A key to achieving accurate and bias-free quantitative monitoring of oxygen and metabolites is to design highly sensitive probes that may be deployed in non-invasive or minimally invasive fashion. Needle-based electrochemical and optical sensors have been developed for in vivo applications [10,11]; while useful, these are still invasive, only usable for a short time, and during that time they are affected by the host response due to the tissue trauma and associated inflammatory/immune response at the site of measurement. In contrast, phosphorescent oxygen-sensing materials are particularly useful in long-term implantable metabolite sensors and can be used by incorporating them in a wide variety of materials [12,13,14]. The phosphorescent oxygen sensor exhibits phosphorescence quenching proportional to the oxygen concentration in the environment and metabolite sensors can be realized by combining these phosphorescent oxygen sensors with oxidase enzyme that consume oxygen during the oxidation of metabolites in the enzymatic reaction.

Phosphorescent oxygen probes carry a unique advantage over electrochemical oxygen sensors as they do not consume oxygen [15]. Sensing dissolved oxygen using phosphorescence is possible using different measurement strategies, including intensity, ratiometric intensity, and phosphorescence lifetime [16,17,18]. In direct and ratiometric phosphorescence intensity-based oxygen measurements, the concentration of the probe and dissolved oxygen cannot be confirmed at the same instance [19,20]. Lifetime-based oxygen measurement is a versatile technique as it is an absolute measurement of intrinsic molecular property, and is independent of the probe concentration [21]. However, the use of phosphorescence quenching for lifetime-based oxygen determination in biological systems has faced formidable challenges since it is hard to combine high sensitivity with biocompatibility [22]. Though dendrimeric phosphors are available in glutamic acid and other biocompatible formulations, the approach to use them in biosensors suffers from limited loading efficiency, limited accessibility of the central probe to the environment, and poor control over uniform spacing among the probes [23,24]. Hence, a phosphorescence lifetime-based oxygen sensor that is biocompatible, stable, highly sensitive, and scalable is a desirable tool for biomedical research, and there has been persistent effort to realize reliable phosphorescence lifetime-based oxygen sensors [16,25,26]. Previous efforts on designing hybrid nanoparticle-based biosensors have been largely confined towards evaluation of matrix stability [27].

In this study, we present a design strategy to realize stable, biocompatible phosphorescent oxygen nanoprobes with high sensitivity and wide dynamic range by encapsulating palladium(II)meso tetra(4-carboxyphenyl)tetrabenzo-porphyrin) (PdBP) in polymeric nanoparticles. We compared properties such as size distribution, sensitivity to oxygen, and toxicity towards 3T3 fibroblasts for different polymeric carriers and surfactants used in synthesis. Further, we examined embedding the nanoprobes along with model enzymes into hydrogel matrices to form nanoparticle-in-microparticle (NIMs) systems and studied their response to metabolite challenges in vitro under simulated physiological conditions to assess potential for continuous monitoring using phosphorescence lifetime readout. These studies identified key characteristics relating chemical and physical properties to function of oxygen-sensitive material and revealed important design considerations for preparation of materials intended for contact with living systems.

## 2. Experimental Section

### 2.1. Materials

Alginate (Cat No. A2158, 75000-100000 Da), ethyl cellulose (EC, Cat No. 200697, 48% ethoxy), polystyrene (PS, Cat No. 182427, 280000 Da by GPC), calcium carbonate (Cat. No. 239216), TRIS base (Cat No. T1503), [2-(Methacryloyloxy)ethyl]trimethylammonium chloride (TMA, Cat. No. 408107, 80% in water), catalase (CAT, from bovine liver, Cat. No. C9322), calcium chloride (Cat No. 22350), pluronic F 68 (PF68, Cat. No. P1300), cetyltrimethylammonium bromide (CTAB, Cat. No. H9151), sodium dodecylsulfate (SDS, Cat. No. L4390), and tetrahydrofuran (THF, Cat No. 401757) were purchased from Sigma-Aldrich, Inc., St. Louis, MO, USA. Trizma hydrochloride (TRIS HCl, Cat No. VWRB85827) and bis-acrylamide (Cat No. 0172, Amresco) were purchased from VWR Chemicals LLC, NY, USA. 2-(methacryloxy)ethylphosphorylcholine (MPC, Cat. No. M2005), glucose oxidase from Aspergillus niger (GOx, Cat No. G0050), (Z,Z,Z)-sorbitan tri-9-octadecenoate (SPAN 85, Cat. No. S0064), polyoxyethylenesorbitan trioleate (TWEEN 85, Cat. No. T0547) were purchased from Tokyo Chemical Company Co., LTD, Tokyo, Japan. 2-hydroxyethylmethacrylate (HEMA, Cat No. 04675, opthalmic grade) was purchased from Polysciences Inc. Warrington, PA, USA. Palladium (II) meso-tetra(4-carboxyphenyl)tetrabenzo-porphyrin) (PdBP, Cat No. T13343) was purchased from Frontier Specialty Chemicals, Logan, UT, USA. Iso-octane (Cat No. 94701) was purchased from Avantor performance materials, LLC, Randor, PA, USA. All of the above chemicals were used as obtained without any further purifications. 1X Dulbecco’s Modification of Eagle’s Medium (DMEM; with L-glutamine, 1 g L^−1^ glucose, and sodium pyruvate) was procured from Corning/Mediatech, Inc., Manassas, VA, USA. Heat-inactivated, fetal bovine serum (FBS) was obtained from Gibco, Grand Island, NY, USA while 100X EmbryoMax^®^ penicillin/streptomycin (P/S) antibiotics and cell counting kit (CCK-8; Cat. No. 96992) for cytotoxicity assay were purchased from Millipore Sigma, St. Louis, MO, USA. Lactate oxidase (LOx, Cat. No. L-1175-1KU) was purchased from AG Scientific, San Diego, CA, USA. Cyanine5.5 N-hydroxysuccinamide ester (CY5.5-NHS ester, Cat. No. BP-22537) was purchased from Broadpharm, San Diego, CA, USA.

### 2.2. Measurements and Instrumentation

The NP’s suspensions were diluted 10 times in deionized water, then drop-casted on carbon-coated copper grids and dried under ambient conditions for transmission electron microscopy (TEM). A JEOL 1200 EX TEM equipped with tungsten filament source at 100 kV acceleration voltage was used for TEM studies. Nanoparticle size distribution in suspensions was determined using the NanoSight LM10 HS nanoparticle tracking analysis (NTA) instrument. Confocal fluorescence microscopy was performed on Olympus FV1000 confocal microscope using 60×/1.2 water immersion objectives. An S25N18G dispersing tool, IKA T25 easy clean digital homogenizer was used to prepare alginate microspheres. A Nexcelom Cellometer Mini device was used for determination of size distribution of alginate microparticles.

For oxygen sensitivity (Stern–Volmer) studies, the hydrogel sample punches were held in place on an optically transparent acrylic sample holder using rubber cantilevers. The acrylic holder with samples was then sealed inside a rectangular flow cell with optical readout windows. Oxygen concentration was varied by mixing air and nitrogen in defined ratios between 0 and 21% using a pair of digitally controlled mass flow controllers, using MKS instruments PR4000B controller to achieve dissolved oxygen levels between 0 and 258 µM. The air–nitrogen mixtures were bubbled into the TRIS buffer in a round-bottomed flask in an incubator and the buffer was circulated over the samples at 37 °C in the flow cell using a recirculating flow system described previously (see Figure S1 for experimental set up) [28]. For modulating the concentration of glucose and lactate for sensitivity analyses, a pair of peristaltic pumps connected to the reservoir of stock solutions and controlled by a custom LabVIEW program was used (see Figure S2 for experimental set up). For lifetime reading, an established time-domain phosphorescence lifetime reader system with 630 nm excitation and 800 nm emission recorded the lifetime of each hydrogel sensor at 10 s intervals as the oxygen, glucose, and lactate concentrations were varied [29].

### 2.3. Synthesis of NPs Containing Oxygen-Sensitive Phosphor

**Figure 1:**
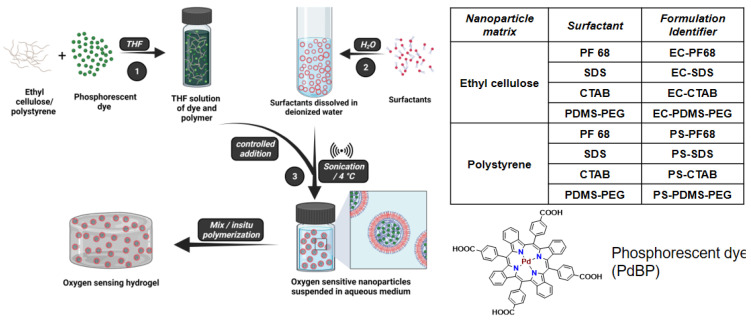
Synthesis of oxygen-sensing nanoparticles. Oxygen-sensitive phosphorescent dye and polymer (ethyl cellulose or polystyrene) are dissolved in tetrahydrofuran (THF). This solution is then mixed with the surfactant solution in deionized water (containing the micelles) during sonication. Nanoparticles were obtained after removal of solvent by evaporation.

We used a nano emulsion technique to prepare oxygen-sensitive NPs as illustrated in Figure 1. In a typical synthesis of nanoparticles, 100 mg of the polymer (EC or PS) was dissolved in 5 mL of THF on a magnetic stirrer by overnight mixing. An amount of 2 mg of PdBP was dissolved into 1 mL of the polymer solution in THF using sonication for 30 min. The above THF solution with dye and polymer was filtered through a 0.2 µ PTFE syringe filter and stored in a glass vial. In a separate 50 mL centrifuge tube, 100 mg of surfactant was dissolved in 20 mL of deionized water using sonication and filtered through a 0.2 µ PTFE syringe filter. Next, the aqueous solution of surfactant was sonicated using a probe sonicator in an ice bath. The THF solution with polymer and dye was injected into the aqueous solution of surfactant within 30 s during sonication and the mixture was further sonicated for 2 min. Finally, the resulting green suspension of NPs was filtered through a 100 µm nylon filter to remove large precipitates, and the volume was reduced to 3.0 mL using centrifuge filtration (MWCO 100 KDa, 3500 RMP). The nanoparticle suspension was washed with 15 mL (3.0 mL × 5 times) of nanopure water in the same centrifuge filter to remove excess surfactants before use. The NPs were stored in vials of 16.6 mg mL^−1^ at room temperature air and protected from light. The above process was repeated for all NPs with different polymer–surfactant combinations. The NP samples were characterized for size distribution using TEM and NTA analyses.

### 2.4. Preparation of Calcium Alginate Hydrogel Slab with Oxygen-Sensitive NPs

For preparation of calcium alginate hydrogel, 75 µL of nanoparticle suspension (1.25 mg NPs) was mixed with 25 µL of 33.3% aqueous suspension of calcium carbonate and 200 µL of 3.0% aqueous solution of sodium alginate. Finally, 100 µL of MES buffer with pH 6.1 was added and the whole mixture was quickly transferred into a rectangular mold made with microscope slides and 0.75 mm Teflon spacer. The release of calcium from the carbonate induced gelation of the alginate, which was allowed to proceed for 20 min. The resulting oxygen-sensing alginate hydrogel slab was carefully recovered and stored for 24 h in TRIS buffer (pH 7.2) with 10.0 mM calcium chloride at 4 °C before testing.

### 2.5. Preparation of Poly(2-methacryloyloxyethylphosphorylcholine) (MPC) Hydrogels with NPs

In a typical synthesis of MPC hydrogel, 91.5 mg of MPC (Mol. Wt. 295.27 g mol^−1^) and 8.0 mg of N,N′-Methylenebisacrylamide (BIS) (Mol. Wt. 154.17 g mol^−1^) were dissolved in 406 µL of 50 mM TRIS buffer. The above mixture was mixed with 94 µL of nanoparticle suspension (1.56 mg of NPs). Next, 12.0 µL of 10.0 mM aqueous solution of ammonium persulfate (APS) and 2.0 µL of N,N,N′,N′-Tetramethyl ethylenediamine (TEMED) were added. Finally, the solution was bubbled with nitrogen for 30 s and quickly transferred into a rectangular sandwich mold with a 0.75 mm thick Teflon spacer. The above solution was placed in a nitrogen environment for 4 hours to allow crosslinking. The hydrogel was then washed in fresh 10 mM TRIS buffer containing 10.0 mM calcium chloride for 12 h and stored in the fresh TRIS buffer solution at 4 °C.

### 2.6. Preparation of Poly(2-hydroxyethylmethacrylate) (HEMA) Hydrogels with NPs

For preparation of HEMA hydrogel, 68.4 mg of 2-hydroxyethyl methacrylate (mol. wt. 130.14 g mol^−1^, d = 1.07 g mL^−1^), 12.4 mg of acrylamide (mol. wt. 70.08 g mol^−1^), and 19.08 mg BIS (Mol. Wt. 154.17 g mol^−1^) were dissolved in 406 µL of 50 mM TRIS buffer by sonication. Next, 94 µL of nanoparticle suspension (1.56 mg of NPs) was added and mixed to obtain a homogenous mixture. To the above solution, 12.0 µL of 10.0 mM aqueous APS and 2.0 µL of TEMED were added, mixed and bubbled with nitrogen for 30 s. Finally, the reaction cocktail was transferred into a sandwich mold, as mentioned for other hydrogels, and placed under nitrogen environment for 4 h to allow complete crosslinking. After 4 h of reaction, the hydrogel was recovered, washed with fresh 10 mM TRIS buffer, and stored under TRIS buffer with 10 mM Calcium chloride at 4 °C.

### 2.7. Preparation of Poly [2-(methacryloyloxy)ethyl]trimethyl-ammonium (TMA) Chloride Hydrogel with NPs

First, 11.6 mg of BIS was dissolved in 295.75 µL of 50 mM TRIS buffer and mixed with 110.5 µL of 80% aqueous solution of 2-(Methacryloyloxy)ethyl]trimethylammonium chloride (mol. wt. 207.70 g mol^−1^). Then, 94 µL of nanoparticle suspension (1.56 mg NPs) was added to the mixture and vortexed. Before polymerization, 12.0 µL of 10.0 mM aqueous APS and 2.0 µL of TEMED were added and nitrogen gas was bubbled, and the solution was transferred into a mold. Finally, the reaction mixture in the mold was placed under nitrogen for 4 h to allow crosslinking, washed with TRIS buffer containing 10.0 mM calcium chloride and stored in the same fresh buffer at 4 °C.

### 2.8. Preparation of Alginate Microspheres Containing Oxidase Enzyme and Oxygen-Sensitive NPs

A homogenous mixture was obtained by mixing 3.75 mL of 4.0% aqueous solution of sodium alginate, 1.25 mL of nanoparticle suspension (5.0 mg NPs) were mixed for 30 min in a nutator to obtain a homogeneous mixture. In a separate container, oxidase enzyme (58.5 mg (2340 U) of glucose oxidase (GOx) or 25 mg (1000 U) of lactate oxidase (LOx)) along with 91.5 mg (>915,000 U) of catalase (CAT) were dissolved in 2.5 mL of 50 mM TRIS buffer by gentle nutation. The alginate solution with NPs and the enzyme solution were mixed for 20 min to obtain the precursor solution. For emulsification, the precursor solution and 10.8 mL of isooctane containing 290 µL of SPAN 85 were mixed at 8000× *g* using a homogenizer (see measurements and instrumentation). Next, 1.5 mL of isooctane containing 140 µL of TWEEN 85 was added while the above mixture was still mixing at 8000× *g*. Stirring was continued for 10 s to form a uniform emulsion, after which 4 mL of 10% *w/w* calcium chloride solution was added and the mixture was stirred for 15 s. Next, the obtained emulsion was transferred into a 500 mL round bottom flask and mixed at 300× *g* using a magnetic stirrer for 20 min to allow the crosslinking of alginate microparticles. The microemulsion was then centrifuged at 2000× *g* and washed twice with deionized water. Finally, particles were subjected to successive centrifugation cycles after mixing with 2% *w*/*w* solutions of PSS or PAH to form layers of alternating charge, as reported previously [30]. The process of depositing one bilayer was repeated 25 times to obtain a ~100 nm-thick [31] film for diffusion control of analyte in the microparticles. Size distribution of alginate microparticles was determined using Cellometer. For fluorescence imaging of the glucose- and lactate-sensing microparticles, we used Cy5.5-labeled PAH solution to form the same number of bilayers on the surface of the microparticles (Supplementary Materials Section S1).

### 2.9. Preparation of Glucose- and Lactate-Sensing Alginate Hydrogels

About 8.8 mg of glucose- or lactate-sensing alginate microparticles were washed twice with 1 mL of deionized water and centrifuged at 6000× *g* for 15 s to isolate the microparticles as a pellet. The microparticles were mixed in 75 µL of nanoparticle suspension on a vortex mixer. To the above mixture, 25 µL of 33.3% aqueous suspension of calcium carbonate and 200 µL of 3.0% aqueous solution of sodium alginate were added and vortexed for 60 s. Finally, 100 µL of MES buffer with pH 5.5 was added, mixed, and the whole mixture was quickly transferred into a rectangular sandwich mold made with microscope slides and 0.75 mm Teflon spacer and allowed to crosslink for 20 min at room temperature under ambient condition. The glucose sensing alginate hydrogel slab was carefully recovered and stored for 24 h in TRIS buffer at pH 7.2 with 10.0 mM calcium chloride at 4 °C before testing.

For control experiments, a free PdBP dye concentration of 5.66 × 10^−5^ M was used to replicate the feed concentration of dye used in the nanoparticles’ preparations. Thus, the concentration of free dye dispersed in hydrogel resembles the concentration of dye in nanoparticles loaded hydrogel sensors.

### 2.10. Characterization for Oxygen Sensitivity

Measurements of phosphorescence lifetime under different oxygen concentrations were performed to determine oxygen sensitivity. The 4.0 × 0.75 mm discs of hydrogel loaded with sensing microdomains were loaded on optically transparent acrylic sample and loaded inside the flow cell. Oxygen concentrations were varied by mixing air and nitrogen in defined ratios between 0 and 21% mass flow controllers to achieve dissolved oxygen levels between 0 and 257.9 µM (see measurements and instrumentation section for details). This range covers the expected average subcutaneous tissue oxygen partial pressure of 52 ± 10 mm Hg (equivalent to dissolved oxygen concentration of 17.6 ± 3.4 µM) in humans [32].

The lifetimes of the samples were recorded at 10 s interval as the oxygen leves were varied [29]. Finally, the Stern–Volmer constant of each hydrogel sensor punch was calculated according to Equation (1) [33]:

*τ*_0_/*τ* = 1 + *K_SV_* [O_2_] (1)

where *τ_0_* is the phosphorescence lifetime in the absence of oxygen and *τ* is the lifetime at a given oxygen concentration [O_2_]. *K_SV_* is the Stern–Volmer constant representing the slope of the linear relationship between the ratio *τ_0_*/*τ* and [O_2_]. *K_SV_* was determined for each sample as the slope of the least-squares regression line for *τ_0_*/*τ* plotted against oxygen.

### 2.11. Glucose and Lactate-Sensing Experiments

To study the glucose and lactate-sensing characteristics, the hydrogel sensors were sealed inside a flow cell as mentioned above for the oxygen sensitivity analysis. Then, glucose or lactate concentration was systematically modulated to obtain the sensitivity data (see measurements and instrumentation section for details). For glucose sensing experiment, stock solutions of 400 mg dL^−1^ glucose in TRIS buffer (pH 7.2) and “blank” TRIS buffer (pH 7.2, no glucose) were mixed at desired proportions and circulated through the flow cell containing the glucose sensors. Similarly, for the lactate-sensing experiment, a stock solution of 20 mg dL^−1^ lactate solution was used. The phosphorescence lifetime of the glucose sensors and lactate sensors were recorded in similar method as described for the oxygen sensitivity studies.

### 2.12. Cytotoxicity Studies of NPs and Alginate Microparticles

NIH/3T3 (mouse embryonic fibroblasts) cells at passage 24 were grown in DMEM culture media supplemented with 10% FBS and 1X concentration of P/S antibiotics. Cell cultures were maintained in a humidified incubator with 5% CO_2_ supply at 37 °C. A cytotoxicity assay was performed according to the manufacturer’s protocol. Briefly, upon achieving 80% of cell confluence, fibroblasts were trypsinized and seeded in the 96 well-plates at 5000 cells per well seeding density in 100 µL of DMEM media (10% FBS, 1X P/S). Non-encapsulated NPs were tested at concentration regimes similar to our standard microsphere concentrations dispersed within the hydrogel matrices (22 mg mL^−1^), which also equates to 4 mg mL^−1^ of microspheres under 10 uL of incubation volume. Therefore, exposure concentration regimes of non-encapsulated NPs, starting from the highest (4 mg mL^−1^) to its 1:10 dilutions were tested. For NPs encapsulated within microspheres, an appropriate exposure dose was first determined to avoid overwhelming cells with excess volume of microspheres coagulating at the bottom of the well. For this, we first calculated the estimated highest concentration of encapsulated NPs dispersed within the hydrogel matrix for any given sensor shape and dimension. Next, we designed our exposure dose regimes for encapsulated NPs starting from the highest (0.117 mg/mL) to 1:8 dilution and tested against the cells (more under Section 3). Cells were grown for 24 h in the incubator and CCK-8 reagent was added to sample wells, followed by absorbance measurement at 450 nm.

## 3. Results and Discussion

We aimed to design a biocompatible, scalable, customizable, and sensitive oxygen sensor. We found that both PS and EC have been extensively used in the design of sensitive oxygen probes [25,34]. In addition, both EC and PS NPs have been studied extensively for their in vivo pharmacokinetics [34,35,36]. This motivated us to explore EC and PS matrices for preparation of NPs impregnated with oxygen sensitive phosphor, PdBP. Since PdBP is known to be a hydrophobic dye, we predicted that the phosphor can be favorably encapsulated in hydrophobic matrix of EC and PS. This further can be beneficial since the interaction of PdBP and environment is minimized, while the oxygen diffusion is retained by the matrix of EC and PS to favor a media-independent oxygen sensitivity. For scalable preparation of the oxygen sensitive NPs, we adopted a nano emulsion technique as illustrated in Figure 1.

We started by determining the solubility of PdBP in THF to be 2.0 mg mL^−1^ (0.2% *w/v*). We further determined that dissolution of polymers, EC or PS in the 0.2% PdBP-THF solution did not lead to precipitation of the phosphor. We therefore used 1:10 PdBP:polymer in the precursor solution to achieve final dye concentration of 0.2% (*w/v*) and polymer concentration of 2.0% (*w/v*). The low concentration of PdBP was used to enhance aggregation-free encapsulation of the dye. Aggregation of phosphors is detrimental to oxygen-sensing systems, as it causes static quenching, formation of long-lifetime species, and consequently limits the dynamic lifetime range of a phosphor in response to oxygen [37,38]. For efficient emulsification we used 20 mL of water containing 0.5 % *w/v* of surfactants. The evaporation of THF was done under inert conditions to prevent oxidation of PdBP [39]. Following a similar protocol, we synthesized EC and PS NPs impregnated with PdBP and tested each matrix with four different surfactants.

Thus, we prepared a total of eight different nanoparticle suspensions with combination of two polymers and four different surfactants, namely: Pluronic F68 (PF68), sodium dodecylsulfate (SDS), hexadecyltrimethylammonium bromide (CTAB), and polydimethylsiloxane-polyethylenoxide (PDMS-PEG). Our attempts to prepare similar suspensions of EC and PS using other candidate surfactants (Tween 40 and polyvinyl alcohol) resulted in unstable formulations and were not pursued further. The eight different formulations of nano emulsions were further studied to evaluate key properties and identify the optimal combination of polymer matrix and surfactant for biosensing applications. The prepared NPs were generally found to be stable in aqueous solution at ambient conditions. However, the nanoparticle suspensions were stored in the dark to avoid potential bleaching or other potential photo-induced effects.

### 3.1. Physical Characteristics of NPs

**Figure 2:**
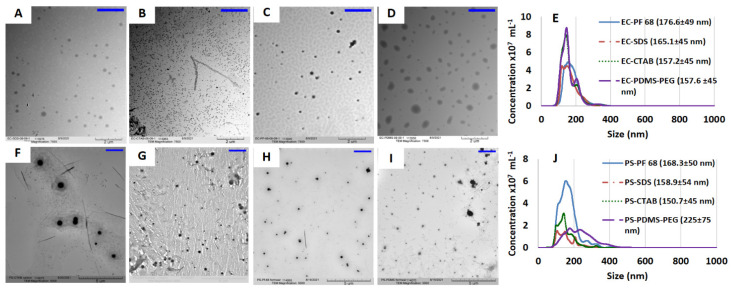
Transmission electron micrograph of ethyl cellulose nanoparticles stabilized with. SDS (EC-SDS) (**A**), CTAB (EC-CTAB) (**B**), PF-68 (EC-PF68) (**C**), and PDMS-*b*-PEG (EC-PDMS-PEG) (**D**). (**E**) NTA analysis results of ethyl cellulose nanoparticles with average size in legend. Polystyrene nanoparticles stabilized with: SDS (PS-SDS) (**F**), CTAB (PS-CTAB) (**G**), PF-68 (PS-PF68) (**H**), and PDMS-*b*-PEG (PS-PDMS-PEG) (**I**). A blue scale bar is provided in the top right of panels A–D and F–I for clarity and it represents 2.0 µm. EC-CTAB nanoparticles were found to be much smaller than all other nanoparticles and (**J**) NTA analysis results of polystyrene nanoparticles with average size displayed in the legend.

Each of the nanoparticle formulations was immediately characterized for size distribution in both solution state and after drop-casting the nanoemulsion. As we envisioned that the oxygen sensitive NPs would be utilized in application to metabolite sensors, narrow and consistent size distribution is important to further encapsulation of the NPs within 9–12 µm microspheres. The TEM images of the NPs were obtained after drop-casting the suspensions on carbon coated copper grids (Figure 2A–D,F–I). TEM shows obvious small particle size (diameter ~100 nm) for EC-CTAB NPs compared to all other EC NP formulations. However, the NTA indicates the average size of all four the EC NPs to be within 157.2–176.6 nm (Figure 2D). Similarly smaller sized particles with diameter of ~100 nm were observed on the TEM micrograph of PS-CTAB NPs while the NTA of PS NPs showed a size range of 150.7–225 nm. The observed smaller particles for EC-CTAB and PS-CTAB NPs is in agreement with the trend in NTA analysis. The absolute difference in size between TEM and NTA is attributed to the fact that NTA measurements track hydrated particles in solution, while the NPs are dried and even undergo some degradation under electron beam irradiation in TEM conditions [40]. We employed NTA, which uses a time-lapse imaging technique to determine the size distribution of particles based on Brownian motion [41]. Figure 2E,J display the size distribution result from NTA analysis of EC and PS NPs, respectively. Generally, the EC NPs had narrower size distributions in aqueous suspension. The EC NPs showed only small change in size distribution with varying surfactants, with only 19 nm difference between the smallest and largest average sizes which were obtained from EC-CTAB (157.2 ± 45 nm) and EC-PF68 (176.6 ± 49 nm), respectively. In contrast, the size of PS particles varied much more dramatically among surfactant types, with a minimum for PS-CTAB (150.7 ± 45nm) and maximum for PS-PDMS-PEG (225 ± 75 nm).

### 3.2. Oxygen Sensing Property of NPs

**Figure 3:**
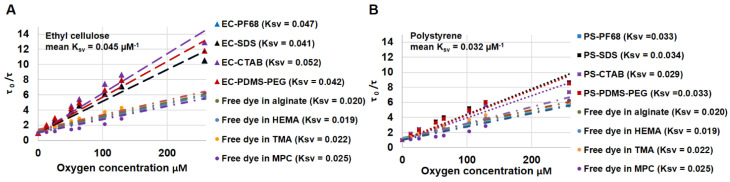
Comparison of oxygen sensitivity for nanoparticles stabilized with different surfactants. Panel (**A**) contains the Stern–Volmer plot for ethyl cellulose NPs dispersed in alginate. Panel (**B**) shows the Stern–Volmer plot for polystyrene NPs dispersed in alginate. The Stern–Volmer constant (K_sv_) of each formulation of NPs is given in the legend within parenthesis (unit for K_sv_ is µM^−1^).

The EC NPs, PS NPs and free PdBP dye dispersed in alginate hydrogels were evaluated for oxygen sensitivity under identical conditions, with results presented in Figure 3 and summarized in Table 1. The Stern–Volmer plots revealed significant differences in sensitivity to oxygen based on the nanoparticle matrix material and the applied surfactant. We found that the average Stern–Volmer constant (*K_sv_*) of EC NPs (0.045 µM^−1^) is 40.4% higher than the mean *K_sv_* of PS NPs (0.032 µM^−1^). This is attributed to the apparent better dispersion of dye within the EC matrix, as well as higher oxygen diffusivity in EC relative to the PS matrix. A better dispersion of dye in a polymer matrix increases accessibility to oxygen, and more rapid oxygen diffusion increases the rate of interaction for a given oxygen concentration [42]. Together, these effects result in increased quenching and wider lifetime range of the phosphor when encapsulated in EC. The average lifetimes of phosphors in EC at 257.9 and 0 µM dissolved oxygen concentrations were 20.3 and 231.5 µs, respectively. For PS NPs, average observed lifetimes at 257.9 and 0 µM dissolved oxygen are 35.5 and 293.4 µs, respectively. Thus, the phosphorescence lifetime changed by a factor of ~11× for EC NPs and ~8× for PS NPs over the range of 0 to 257.9 µM dissolved oxygen, respectively. It is clear from the above data that the phosphorescence of PdBP is more readily quenched in EC matrix than in PS under equivalent oxygen levels [43]. We attribute this to the matrix polarity and rigidity induced effects on porphyrin dyes [44]. In contrast to the above observations, the free PdBP dye dispersed in the same alginate matrix showed ~55% lower sensitivity than ethyl cellulose NPs and 37% lower sensitivity compared to polystyrene NPs. Clearly, encapsulation within nanoparticles is preferred to direct dispersion in alginate matrix.

### 3.3. Effect of Dispersion Medium on Performance of Oxygen-Sensing Nanoparticle

**Figure 4:**
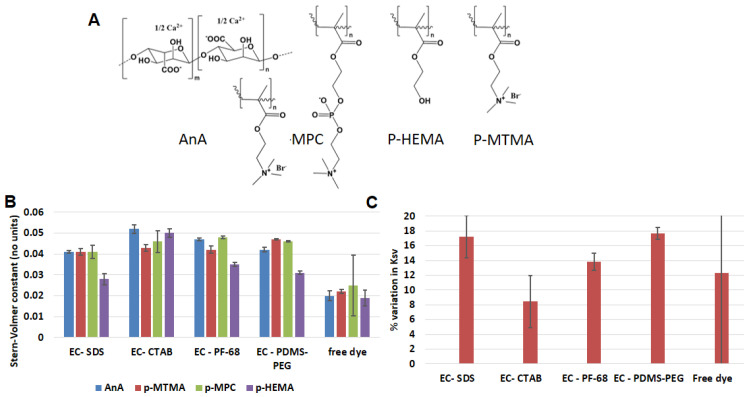
(**A**) Molecular structures of repeat units of calcium alginate (AnA), poly methacryloxyethyl phosphorylcholine (p-MPC), poly hydroxyethylmethacrylate (p-HEMA), and poly trimethylaminoethyl methacrylate (p-MTMA) (**B**) Ksv (µM^−1^) values for different formulations of ethyl cellulose NPs in different matrix and (**C**) percentage variation in K*_sv_* between different hydrogel matrices for different ethyl cellulose NPs.

After determining the best nanoparticle matrix (EC), we proceeded to evaluate the effect of the hydrogel dispersion medium on the oxygen sensitivity of embedded EC NPs. Though highly oxygen-sensitive phosphorescent dyes are known, the major limitation of molecularly dissolved systems is variation in solubility of the dyes in different media [44]. Thus, the dispersion medium is a major factor in influencing the sensitivity of an oxygen-sensing system and hence its applicability. In this study we dispersed EC-PF68, EC-SDS, EC-CTAB, and EC-PDMS-PEG in four different hydrogel matrices. We selected calcium alginate as anionic matrix (AnA), poly [2-(methacryloxy)ethyl]trimethyl-ammonium (p-MTMA) as cationic matrix, poly [2-(methacryloxy) ethyl]phosphorylcholine (p-MPC) was selected as zwitterionic matrix, and poly 2-hydroxylethyl methacrylate was selected as neutral matrix (p-HEMA). All 16 samples from the above combination of NPs and hydrogel matrix were characterized using Stern–Volmer studies. Table 1 summarizes the results effect of different matrix on oxygen sensitivity of EC NPs. EC-CTAB exhibited the highest mean sensitivity to oxygen in all hydrogel matrices, with average Stern–Volmer constant (*K_sv_*) of 0.048 ± 0.004 µM^−1^. The oxygen sensitivity of the EC formulations with different surfactants based on magnitude of *K_sv_* followed the order of EC-CTAB > EC-PDMS-PEG > EC-PF68 > EC-SDS with statistically significant difference (*p* < 0.05) with lowest *K_sv_* value of 0.038 ± 0.006 µM^−1^ for EC-SDS. By comparing the oxygen sensitivity of the different matrices for the same nanoparticle formulation, EC-PDMS-PEG showed a maximum variation in *K_sv_* (17.7%). (Figure 4). In contrast, EC-CTAB exhibited the least variation in *K_sv_* (8.3%) value between hydrogel matrices, followed by EC-PF68 (12.7%), and finally EC-SDS (17.6%) (Figure 4). For further comparison, we dispersed free PdBP dye in all four different hydrogel matrix and evaluated oxygen sensitivity (Table 1).

The global average of *K_sv_* values for EC NPs across all surfactants and dispersion hydrogel matrix is 0.042 ± 0.004 µM^−1^, while the *K_sv_* value for free dye across all hydrogel dispersion matrix (0.021 ± 0.002 µM^−1^) (Table 1) is 50.0% lower with unpredictable changes in values between different hydrogel matrices. This further confirms what was noted above: the encapsulation of dye inside EC NPs is beneficial, in this case improving sensitivity by 100%.

### 3.4. Cytotoxicity Studies of Non-Encapsulated NPs and Encapsulated NPs within Alginate Microspheres

To assess the cytocompatibility of both nanoparticles (NPs; non-encapsulated) and nanoparticles-in-microparticles (NIMs; NPs encapsulated within alginate microparticles), we applied cell viability assays with NIH/3T3 fibroblast cells. For non-encapsulated NPs, we incubated and tested the cells against 1:5 and 1:10 dilutions of the highest concentration of NPs that would be present in the final polymer solution. The calculation for non-encapsulated NPs exposure and incubation was based on a standard concentration of microparticles within the hydrogel matrices used in previous studies (22 mg mL^−1^). This translates to 4 mg/mL concentration of NPs in 10 uL of total incubation volume with the cells. Hence, we incubated and tested cells with three different concentrations of non-encapsulated NPs, starting from the highest concentration (4 mg mL^−1^), 1:5 dilution (0.75 mg mL^−1^) and 1:10 dilution (0.14 mg mL^−1^), respectively.

**Figure 5:**
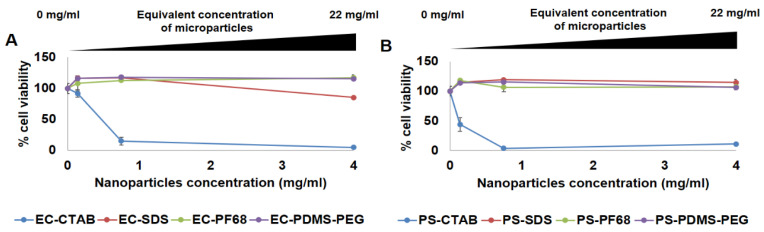
(**A**) % Cell viability in presence of ethyl cellulose NPs. (**B**) **%** Cell viability in presence of polystyrene NPs.

As depicted in Figure 5, the non-encapsulated forms of NPs that used CTAB as the surfactant (both EC-CTAB and PS-CTAB) were found to be highly cytotoxic, with dose-dependent increase in toxicity. This is consistent with previous observations of cytotoxicity for CTAB used as a surface coating on nanoparticles [45]. At the lowest concentration of 0.14 mg/mL, EC-CTAB had relatively low toxicity, whereas PS-CTAB still induced significant cell death (~40% cell viability). However, all other preparations were found to be non-toxic to the cells, with cell viabilities at both lower and higher concentrations around ~100%. An exception to this case was EC-SDS, which showed slightly decreased viability (~80%) at the highest exposure concentration. The lower cytotoxicity of SDS-coated NPs is consistent with previous observations indicating SDS-coated nanoparticles have lower cytotoxicity than CTAB coated nanoparticles [45]. From these observations, we concluded that CTAB surfactants should not be used in any preparations where NPs could be released to contact cells. We then similarly tested the cytotoxity of EC and PS NIMs against the 3T3 fibroblasts. We selected the exposure dose (amount) of microparticles based on the amount that would be dispersed within a given volume of the hydrogel matrix in the final application of these sensors. Specifically, we determined the dose of microparticles dispersed within two commonly used shapes and dimensions of hydrogel matrices that are frequently employed during our in vitro flow cell and in vivo implantation experiments, respectively: a 3D disc [3 mm diameter × 0.75 mm thickness] with volume of 5.29 mm^3^ and a 3D strip [0.5 (*w*) × 0.5 (h) × 5 (l) mm] with a volume of 1.25 mm^3^. Based on our calculations, the highest dose of microparticles dispersed within cylindrical-shaped and strip-shaped sensor punches were estimated to be ~0.12 mg and ~0.03 mg, respectively. Therefore, we designed and tested the dose regimes for NIMs to cover the highest possible dose for each given form factor as well as additional lower doses (1:4 and 1:8 dilution doses for discs and 1:4 dilution dose for strips, respectively).

**Figure 6:**
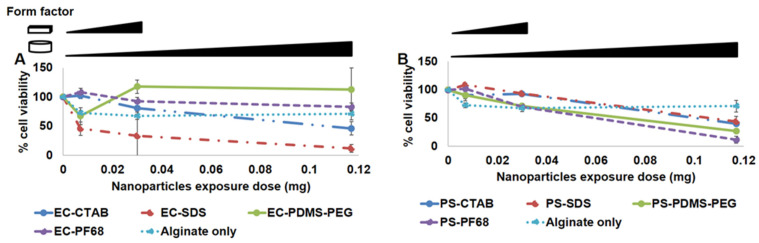
Panel (**A**) Cell viability in presence of ethyl cellulose NPs and (**B**) polystyrene NPs, encapsulated in alginate microspheres.

As depicted in Figure 6, the highest toxicity was observed from the EC-SDS NIMs whereas EC-PDMS-PEG and EC-PF68 NIMs showed negligible toxicity (~100% cell viability). To our surprise, PS-CTAB and PS-SDS NIMs were not toxic to the cells at lower concentrations; however, all four samples containing PS were apparently cytotoxic (<50% viability) at the highest dose (0.12 mg). This trend supports the notion that the EC NPs are less cytotoxic than PS NPs in general, with EC-PF68 and EC-PDMS-PEG stabilized NPs and NIMs demonstrating higher cytocompatibility over other formulations.

### 3.5. Design of NIMs Biosensors Using NPs Encapsulated Microdomains

**Figure 7:**
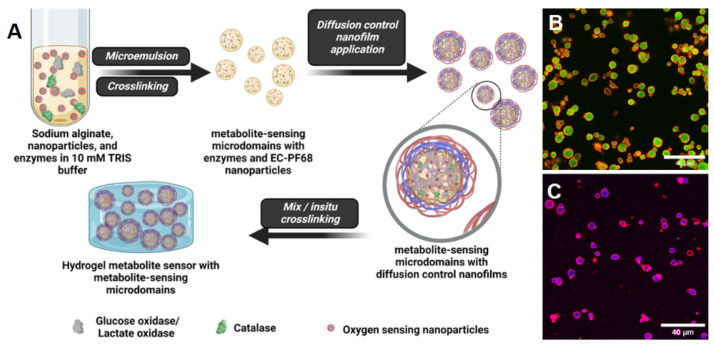
(**A**) Synthesis of glucose and lactate-sensing microdomains encapsulating glucose oxidase or lactic acid oxidase, catalase, and EC-PF68 NPs. The micro-reactors are coated with polyelectrolyte multilayer glucose diffusion control nanofilms by layer-by-layer sequential deposition method. Finally, the microreactors are dispersed in calcium alginate hydrogels to obtain the hydrogel glucose or lactate sensors. Confocal fluorescence composite image of (**B**) glucose sensors with GOx and EC-PF68 NPs in the core (yellow) and diffusion control nanofilms (red) (**C**) Lactate sensors with LOx and EC-PF68 NPs in the core (blue) with fluorescent labeled diffusion control nanofilms (red). Scale bar on panels B and C is 40 µm.

Given the overall combination of high sensitivity (K*_sv_* = 0.043 ± 0.006 µM^−1^) in free form and after encapsulating in microspheres and lowest overall cytotoxicity, we selected the EC-PF68 NPs for further evaluation within metabolite sensors intended as implanted devices. To demonstrate the utility of the EC-PF68 NPs in designing a sensitive metabolite sensor, we encapsulated EC-PF68, GOx (or LOx), and CAT in alginate hydrogel microparticles, as shown in Figure 7 [30]. While our previous work has demonstrated stable and reliable immobilization of enzymes and phosphors within alginate hydrogel microdomains, [46] this is the first example of using PdBP encapsulated within NPs instead of free PdBP dye introduced directly into the microparticle matrix. Thus, the EC NPs are a new sensing element incorporated to form nano-in-micro (NIMs) glucose and lactate sensors for improved sensitivity and dynamic lifetime range. Confocal fluorescence microscope images of the glucose-sensing microdomains made with nanofilms containing Cy5.5-labeled polyallylamine clearly show the diffusion control bilayers (red) attached to the surface of the microspheres and the nanoparticles (green/blue) trapped in the core (Figure 7B,C) [47].

**Figure 8:**
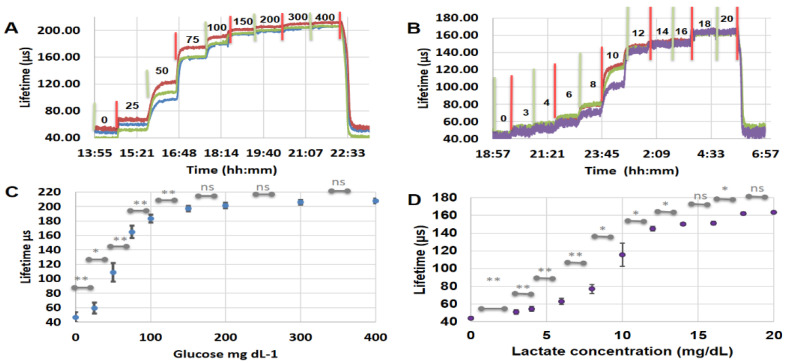
(**A**) Time ramp of glucose response from the hydrogel glucose sensors containing glucose sensitive microdomains, and EC-PF68 NPs, (**B**) The time ramp response of hydrogel lactate sensors with lactate-sensitive microdomains in the range of 0–20 mg dL^−1^ lactate in TRIS buffer. (**C**) A plot of average lifetime of glucose sensors vs. glucose concentration from hydrogel glucose sensors (n = 4). (**D**) Plot of average lifetime of lactate sensors (n = 4) vs. lactate concentration. Each response curve in panels (**A**) and (**B**) represents 3 distinct sensors prepared under identical conditions.

The glucose sensor fabricated using EC-PF68 NPs exhibited fully reversible response to glucose concentration over the range of 0–400 mg dL^−1^ in TRIS buffer (Figure 8). The sensitivity to glucose was found to be 0.40 µs dL mg^−1^ (1.1% dL mg^−1^). The response time to achieve 90% of the steady-state response following a step change in glucose concentration was estimated to be 20 min. This is slightly higher than the response time seen in FRET-based sensors with typical 4–10 min response in 0–200 mg dL^−1^ concentration range, and our previous observations based on HEMA based glucose sensors between 0 and 140 mg dL^−1^ glucose [48,49]. The glucose sensors presented here produce statistically significant signal change at each step change in glucose concentration between 0 and 150 mg dL^−1^. The glucose sensor response of 445% total increase in lifetime at 400 mg dL^−1^ relative to that at baseline (0 mg dL^−1^) is nearly 2× better than previously reported similar microdomain-based sensors based on direct dye loading with 25 diffusion control bilayers [46]. Further, it is 10 times better than the lifetime response observed in agarose beads-based glucose sensors and 40 times better than polystyrene beads based glucose sensors [50]. In comparison with direct dye loaded glucose sensors, the EC NP based sensors have 1.6 times higher lifetime ratio [51]. The reproducibility of the sensor response also improved compared to our previous results, leading to statistically significant response throughout 0–150 mg dL^−1^ glucose [46]. The glucose sensors showed nonlinear response between 0 and 150 mg dL^−1^ glucose with 423% (47 to 197 µs) change in lifetime similar to our previous observation [51,52,53]. The limit of detection (LoD), the maximum distinguishable glucose concentration (MDGC), and glucose sensing range were found to be 13.18 mg dL^−1^, 106.2 mg dL^−1^, and 93.0 mg dL^−1^, respectively, based on a second degree polynomial fit between 0 and 150 mg dL^−1^ glucose (See Figure S6 and Section S2 in Supplementary Materials). These glucose sensors are a demonstration of the utility of the NPs in a model system. It is important to again note that the sensitivity of these glucose sensors can be tuned to match the application by using the thickness of diffusion control nanofilms, enzyme concentration and activity, and particle density in the hydrogel matrix [46,54].

To evaluate the generalizability of these systems, we further prepared and tested lactate sensors by encapsulating lactic acid oxidase in alginate microdomains along with EC-PF68 NPs and catalase enzyme. Here we employed 10 bilayers for effective diffusion control of lactate in the lactate-sensing microdomains in contrast to the 25 bilayers used in glucose sensors, based on observations from previous studies [55]. As with glucose sensing, the use of EC-PF68 NPs instead of free dye inside the lactate-sensing microdomains resulted in many advantages. The lactate sensor showed a sensing range of 11.9 mg dL^−1^, with LoD for lactate determined to be 1.8 mg dL^−1^. The maximum distinguishable lactate concentration (MDLC) was calculated to be 13.7 mg dL^−1^ while the lactate sensitivity was 10.7 µs dL mg^−1^. Thus the sensing range, MDLC, and sensitivity are significantly improved by 29%, 47% and 223%, respectively, compared to our previous report where we directly trapped the phosphor directly in the alginate microdomains [55]. The observed improvement can be attributed to enhanced trace sensitivity of the EC-PF68 encapsulated form compared to the dispersed dye.

The results in the current study compare favorably with other examples from recent published works. Fluorine-functionalized metal complexes in PS NPs were developed for enhanced oxygen sensitivity (*K_sv_* = 0.0026 µM^−1^), [25] nearly 50% lower than our observations in EC NPs. PDMS-PEG NPs with iridium complex that showed 2.5 times enhancement in phosphorescence lifetime between ambient and anoxic conditions, [56] the ethyl cellulose nanoparticles proposed here are 3 times more sensitive with 11 fold increase in lifetime between ambient and anoxic conditions. Platinum tetrabenzoporphyrin embedded in poly(styrene-*block*-vinylpyrrolidone) showed a dynamic lifetime range of 41 µs, [16] and more complicated architectures of NPs with thermoresponsive shells showed maximum dynamic lifetime range of 33 µs, [57] platinum octaethylporphine embedded in polyfluorene NPs showed a dynamic lifetime range of 27 µs [58]. In contrast, the EC NPs in this study possess a dynamic lifetime range of 208 µs, ~11 times enhancement in lifetime between ambient and anoxic conditions, and an average *K_sv_* of 0.042 µM^−1^ (average of different hydrogel matrix and surfactant combined).

Interestingly, most of the reported oxygen sensors that displayed trace oxygen sensitivity are known to be limited by their form factor and mostly used in gas phase. For example, a recently reported open source networkable oxygen sensor with a LoD of 26 ppm (gas phase) used 5,10,15,20-Tetrakis (pentafluorophenyl)-21H,23H-porphyrin palladium (II) coated on a glass disk as the sensing element [59]. An oxygen limit of detection of up to 0.35 ppb (in the gas phase) was observed in 5–10 µm polymeric films loaded with temperature-sensitive ruthenium dye and oxygen-sensitive Fullerene C_70_ for use in gaseous mixtures [43,60,61]. Oxygen ingress in PET containers was measured with sensors in “spot” form (disc) containing phosphorescent oxygen sensitive dye with a detection limit of 1 ppb [62]. Metal organic framework-based trace oxygen sensor supported on electrospun polyacrylonitrile nanofibers had a limit of detection of 14.8 ppm in gas phase; however, the sensitivity rapidly dropped upon exposure to water [63]. Thus, retaining trace oxygen sensitivity in aqueous nano-suspensions along with biocompatibility has been a consistent challenge. In contrast, the EC-PF68 NPs used in this study showed a change of 99 µs in lifetime from 1% (39.2 ppm; lifetime = 122.1 ± 3.5 µs) to 0% (0 ppm; lifetime = 221.0 ± 5.7 µs) oxygen concentrations with a limit of detection of 1.96 µM (6.2 ppm) dissolved oxygen. Thus, EC NPs have unique water-resistant trace oxygen sensitivity as well as biocompatibility, making them very attractive as elements for monitoring oxygen and/or other metabolites in biological systems.

## 4. Conclusions

In this study we have presented the design, optimization, and characterization of oxygen sensitive NPs encapsulating near-infrared phosphors; these are attractive for further incorporation into nano-in-micro (NIMs) metabolite sensors. Based on the high oxygen sensitivity, relatively low impact of matrix on sensitivity, and overall biocompatibility, we were able to successfully conclude that ethyl cellulose NPs coated with PF-68 (EC-PF68) present the best formulation of eight different combinations tested. The synthesized NPs are easily stored as aqueous suspension under ambient conditions, and they are not cytotoxic at realistic concentrations. Further, The high *K_sv_* (average *K_sv_* = 0.042 µM^−1^) values, wide phosphorescence lifetime range (208 µs) between 0 and 258 µM oxygen, and LoD of 0.0016 µM observed for EC NPs makes them one of the most sensitive systems capable of monitoring dissolved oxygen in aqueous media ever reported.

We further evaluated the potential use of EC-PF68 NPs in fabrication of NIMs-based glucose and lactate monitoring sensors, and tested with the physiologically relevant concentrations (70–140 mg dL^−1^ glucose and <2 mM of lactate) [64,65]. The use of EC-PF68 NPs in glucose and lactate-sensing microdomains has some unique advantages. Both glucose and lactate sensors generated statistically significant and reproducible responses, higher sensitivity, wider total lifetime ranges, and lower limits of detection compared with previous reports using molecularly dissolved dye. Thus, the combination of nanoparticle-encapsulated phosphors and our microparticle-based diffusion controlled enzyme microreactor results in modular NIMs with superior performance. While further improvements in the diffusion control of the glucose and lactate can help improve the sensing range of these sensors for specific applications, this study demonstrates the generalizable approach to building enzymatic biosensors.

## Figures and Tables

**Table 1:** The effect of hydrogel matrix on oxygen sensitivity of ethyl cellulose nanoparticles (EC NPs) coated with different surfactants.

Surfactant	pHEMA ^a^ (τ_21%_ (µs)/τ_0%_ (µs)/K_sv_ (µM^−1^))	AnA ^a^ (τ_21%_ (µs)/τ_0%_ (µs)/K_sv_ (µM^−1^))	pMTMA ^a^ (τ_21%_ (µs)/τ_0%_ (µs)/K_sv_ (µM^−1^))	pMPC ^a^ (τ_21%_ (µs)/τ_0%_ (µs)/K_sv_ (µM^−1^))	Average K_sv_ ± S.D. (µM^−1^)
SDS ^b^	24.1 ± 1.9/192.7 ± 7.2/0.028	23.2 ± 0.6/244.0 ± 3.5/0.041	21.5 ± 0.3/236.5 ± 1.4/0.041	23.3 ± 2.1/256.6 ± 5.1/0.041	0.038 ± 0.006
CTAB ^b^	11.3 ± 0.3/153.4 ± 2.0/0.050	17.3 ± 0.2/224.6 ± 11.6/0.052	24.9 ± 0.9/283.7 ± 4.3/0.043	21.9 ± 4.3/236.7 ± 2.4/0.046	0.048 ± 0.004
PF-68 ^b^	18.6 ± 0.3/180.0 ± 3.3/0.035	20.9 ± 0.1/221.0 ± 5.8/0.047	23.0 ± 0.4/257.2 ± 10.6/0.042	19.7 ± 0.4/244.3 ± 6.6/0.048	0.043 ± 0.006
PDMS-PEG ^b^	19.3 ± 0.2/173.3 ± 4.3/0.031	20.0 ± 0.6/236.3 ± 4.1/0.042	21.5 ± 0.1/269.7 ± 5.3/0.047	20.2 ± 0.2/246.1 ± 3.5/0.046	0.041 ± 0.007
Free PdBP	39.5 ± 0.4/220.6 ± 37.9/0.019	41.0 ± 6.3/ 235.9 ± 7.1/0.020	24.9 ± 1.3/150.2 ± 3.5/0.022	31.2 ± 12.6/225.6 ± 13.9/0.025	0.021 ± 0.003

^a^ pHEMA—Poly (2-Hydroxyethyl methacrylate)—Triethylene glycol dimethacrylate hydrogel; ^b^ CTAB—Cetyltrimethylammonium bromide; SDS—Sodium dodecyl sulfate; PF68—Pluronic F68; PDMS-PEG—Poly(dimethylsiloxane-b-ethylene oxide) hydrogel. AnA—Calcium alginate hydrogel; pMTMA—poly [2-(Methacryloyloxy) ethyl]trimethylammonium chloride—Bis-acrylamide hydrogel; pMPC—poly (2-Methacryloyloxyethyl phosphorylcholine)—Bis-acrylamide hydrogel.

## Data Availability

Not applicable.

## Supplementary Materials

The following supporting information can be downloaded at: https://www.mdpi.com/article/10.3390/bios13010141/s1, Figure S1: An illustration of experimental set up used for Stern-Volmer characterization; Figure S2: An illustration of experimental set up used for glucose and lactate sensing experiments; Figure S3: Lifetime response of polystyrene NPs to oxygen. Panel A is PS-SDS, panel B is PS-CTAB, panel C is PS-PF68, and panel D is PS-PDMS-PEG; Figure S4: Lifetime response of ethyl cellulose NPs to oxygen. Panel A is EC-SDS, panel B is EC-CTAB, panel C is EC-PF68, and panel D is EC-PDMS-PEG; Figure S5: Oxygen response of ECNPs with different surfactants dispersed in different hydrogel dispersion medium; Figure S6: The second-degree polynomial fit used for the glucose sensor in 0-150 mg dL-1 glucose concentration; Table S1: Comparison between polystyrene and ethyl cellulose matrix for nanoparticles.
