# Supplementary material for: NIR Luminescent Oxygen-Sensing Nanoparticles for Continuous Glucose and Lactate Monitoring
Source: Biosensors (Basel). 2023 Jan 14;13(1):141. doi: 10.3390/bios13010141 (PMC9855917; doi:10.3390/bios13010141)
Supplement: Supplementary file 1 [file biosensors-13-00141-s001.zip › biosensors-2121551-supplementary.pdf]

Supplementary materials

For

NIR luminescent oxygen-sensing nanoparticles for continuous glucose and lactate monitoring

S1. Preparation of CY5.5 labeled Polyallyamine hydrochloride.

1.8 g of polyallylamine hydrochloride (PAH) was dissolved in 10 mL of deionized water. To the above solution 1.8 mg of CY5.5 NHS ester dissolved in 1 mL of DMSO was added and the mixture was stirred for 12 hours. After 12 hours, the reaction mixture was poured into 200 mL of acetone with mixing. The resulting light blue precipitate was recovered by centrifuging at 4000 rpm for 5 minutes. Finally, the precipitate was dissolved in deionized water and lyophilized to obtain the dry powder of CY5.5 labeled PAH. The CY5.5 labeled PAH was used to prepare the glucose and lactate sensing microspheres for fluorescence imaging.

S2. Calculation of sensor parameters reported in the manuscript.

For calculation of limit of detection (LoD), the standard deviation (positive value only) at 0 mg dL^{-1} of metabolite. Then, the lifetime at LoD was calculated according to the equation**, lifetime at LoD = (lifetime at mg dL^{-1} + (3x standard deviation at o mg dL^{-1})).** Finally, the quadratic equation, $\mathbf{y = -0.0044x^2 + 1.7042x + 46}$ obtained from **Figure S6**. In the quadratic equation the, **Y is the lifetime at LoD and X is the LoD**. The LoD was obtained by solving the quadratic equation.

The maximum distinguishable glucose concentration (MDGC) was calculated by the following equation below. MDCG is the metabolite concentration at a response 10% lower than the response at maximum metabolite concentration.

Lifetime at MDCG = lifetime at maximum metabolite concentration x 0.9

MDCG is finally calculated by substitution the lifetime at MDCG for Y in the quadratic equation $\mathbf{y = -0.0044x^2 + 1.7042x + 46}$**.**

Sensing range of the sensor is calculated by the equation, **Sensing range = MDCG-LoD**

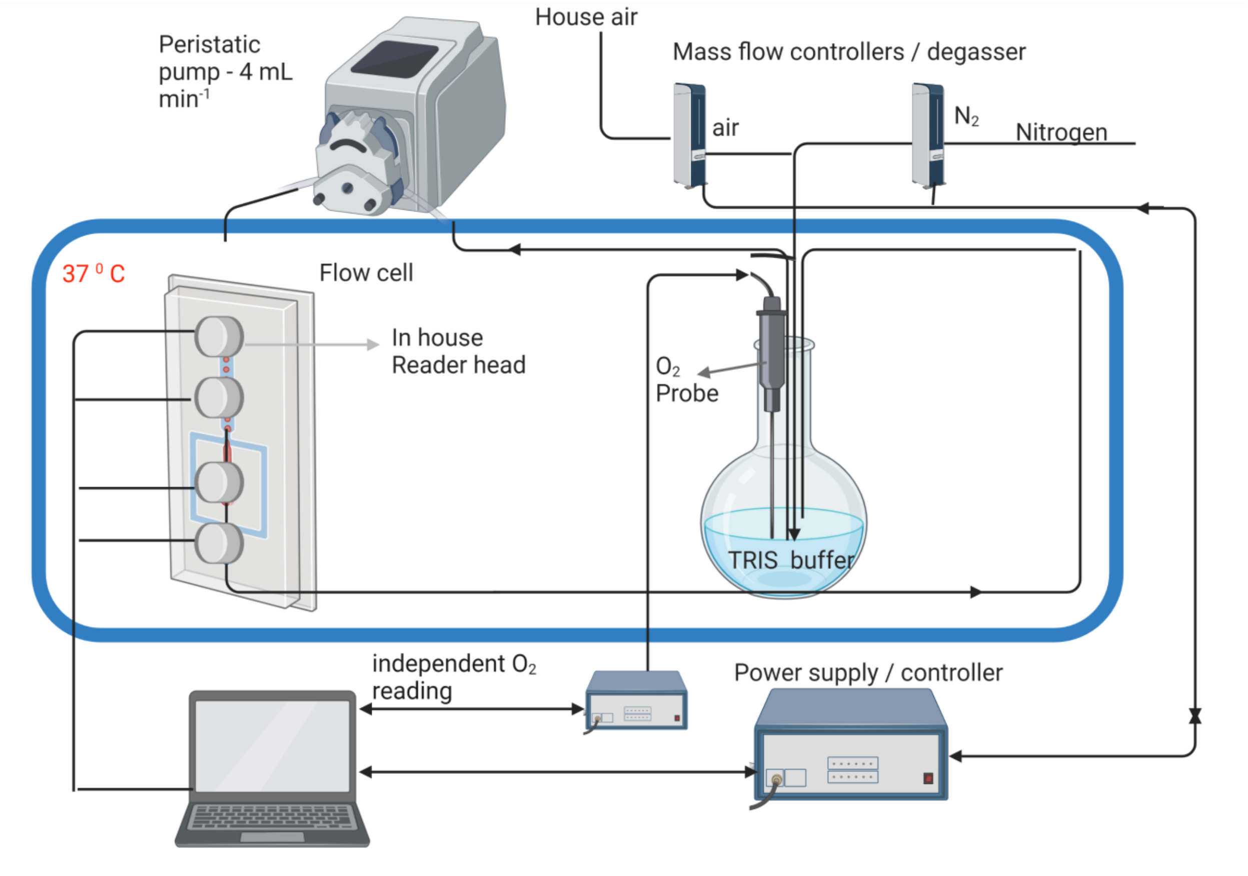

Figure S1. An illustration of experimental set up used for Stern-Volmer characterization.

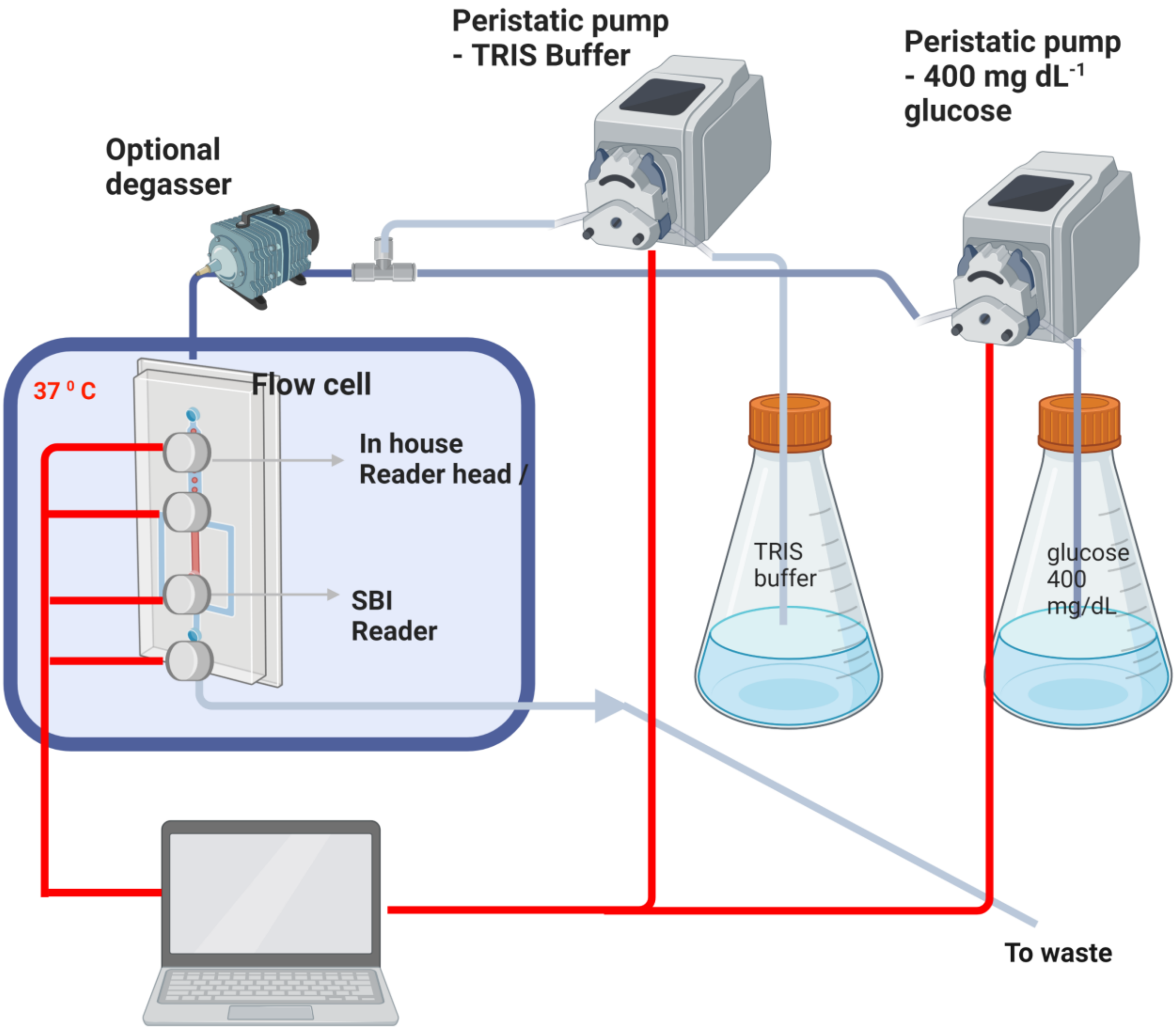

Figure S2. An illustration of experimental set up used for glucose and lactate sensing experiments.

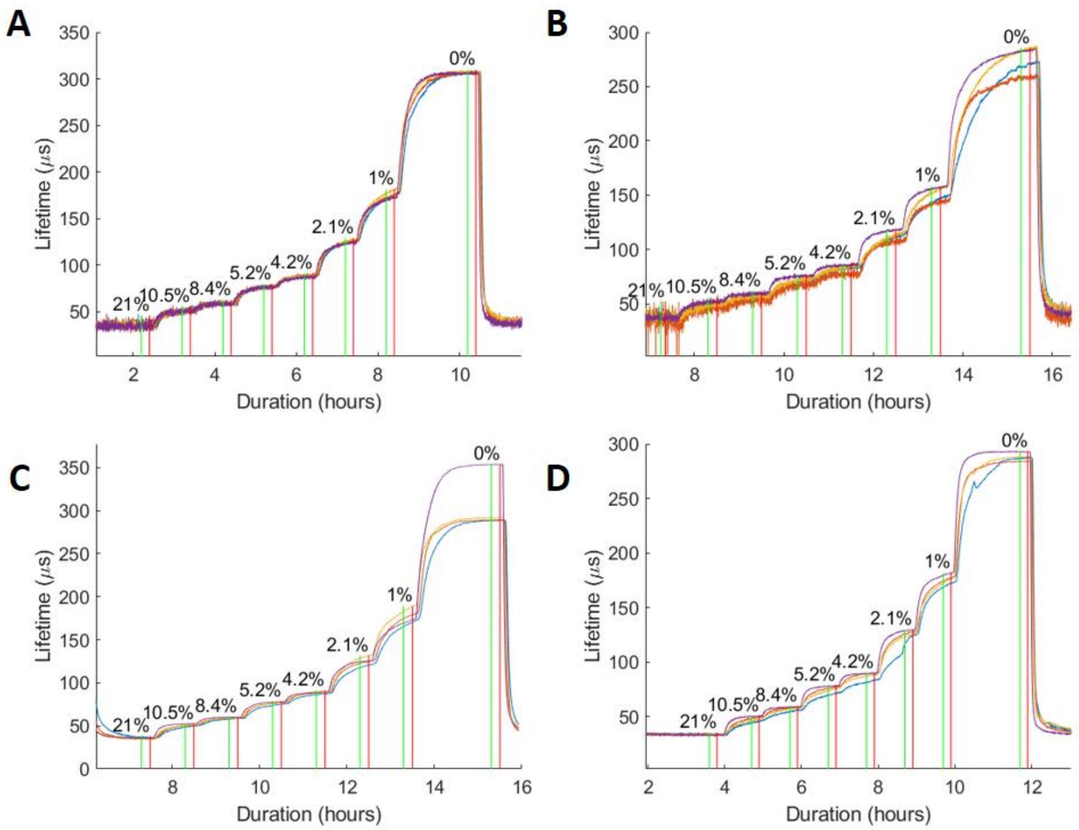

Figure S3. Lifetime response of polystyrene NPs to oxygen. Panel **A** is PS-SDS, panel **B** is PS-CTAB, panel **C** is PS-PF68, and panel **D** is PS-PDMS-PEG. Concentrations of dissolved oxygen at each step can be understood as: 21% (~257.9 µM), 10.5% (~128.95 µM), 8.4% (~103.16 µM), 5.2% (~63.86 µM), 4.2% (~51.58 µM), 2.1 (~25.79 µM), and 0% (0 µM) respectively.

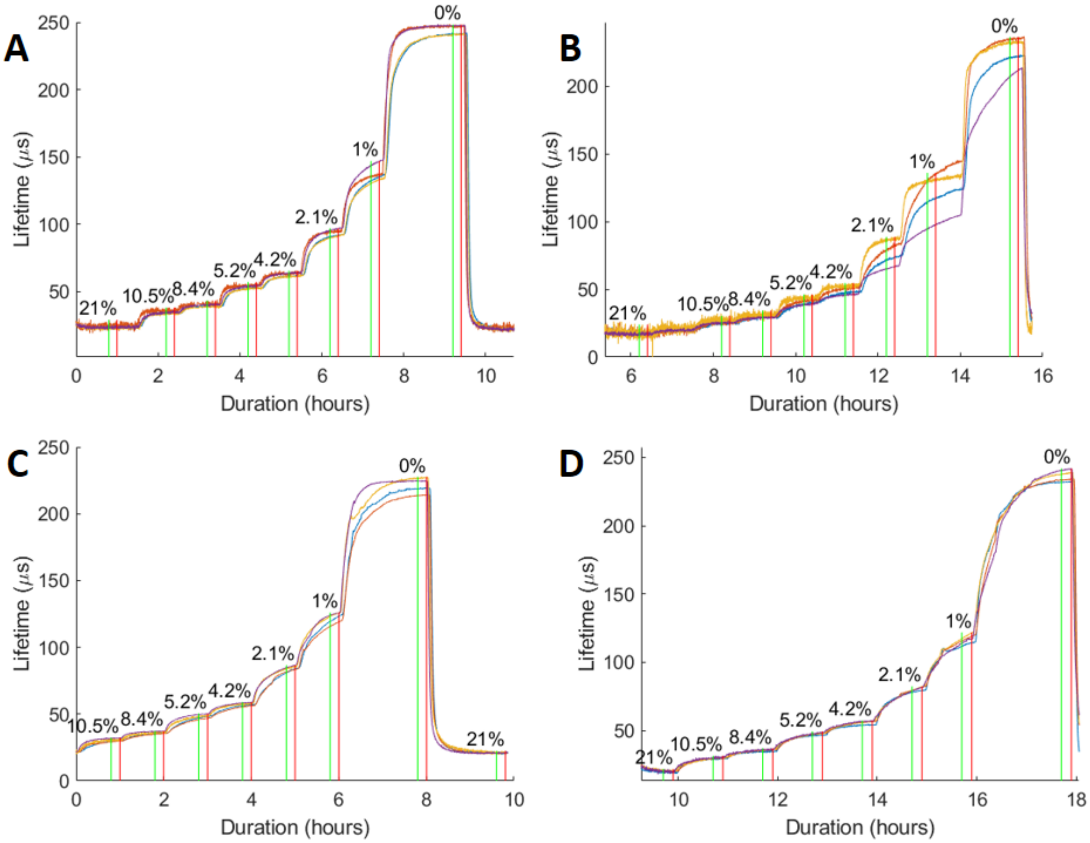

Figure S4. Lifetime response of ethyl cellulose NPs to oxygen. Panel **A** is EC-SDS, panel **B** is EC-CTAB, panel **C** is EC-PF68, and panel **D** is EC-PDMS-PEG. Concentrations of dissolved oxygen at each step can be understood as: 21% (~257.9 µM), 10.5% (~128.95 µM), 8.4% (~103.16 µM), 5.2% (~63.86 µM), 4.2% (~51.58 µM), 2.1 (~25.79 µM), and 0% (0 µM) respectively.

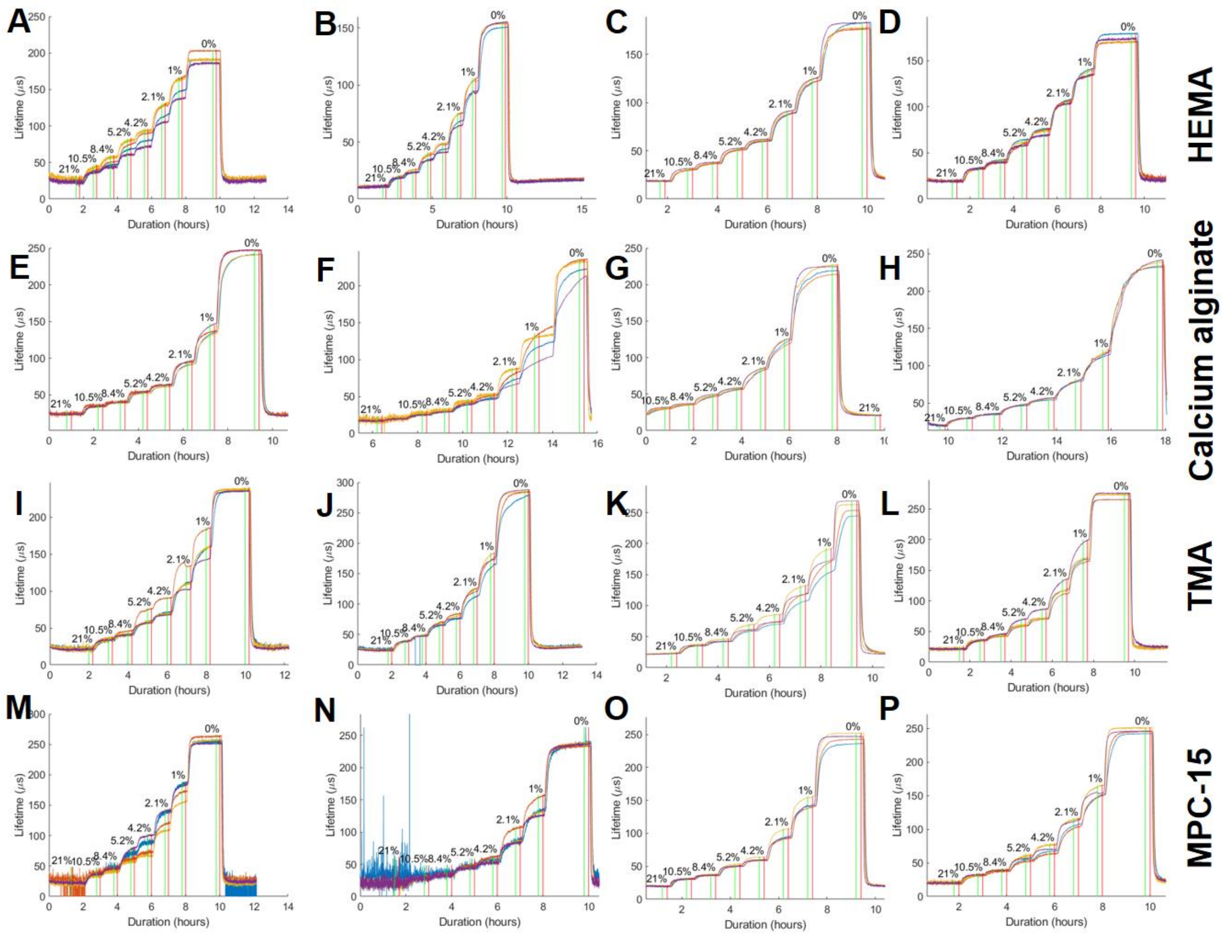

Figure S5. Oxygen response of ECNPs with different surfactants dispersed in different hydrogel dispersion medium. **A** EC-SDS in HEMA**, B** EC-CTAB in HEMA, **C** EC-PF68 in HEMA, and **D** EC-PDMS-PEG in HEMA. **E** EC-SDS in Alginate**, F** EC-CTAB in alginate, **G** EC-PF68 in alginate, and **H** EC-PDMS-PEG in alginate. **I** EC-SDS in TMA**, J** EC-CTAB in TMA, **K** EC-PF68 in TMA, and **L** EC-PDMS-PEG in TMA. **M** EC-SDS in MPC-15**, N** EC-CTAB in MPC-15, **O** EC-PF68 in MPC-15, and **P** EC-PDMS-PEG in MPC-15.

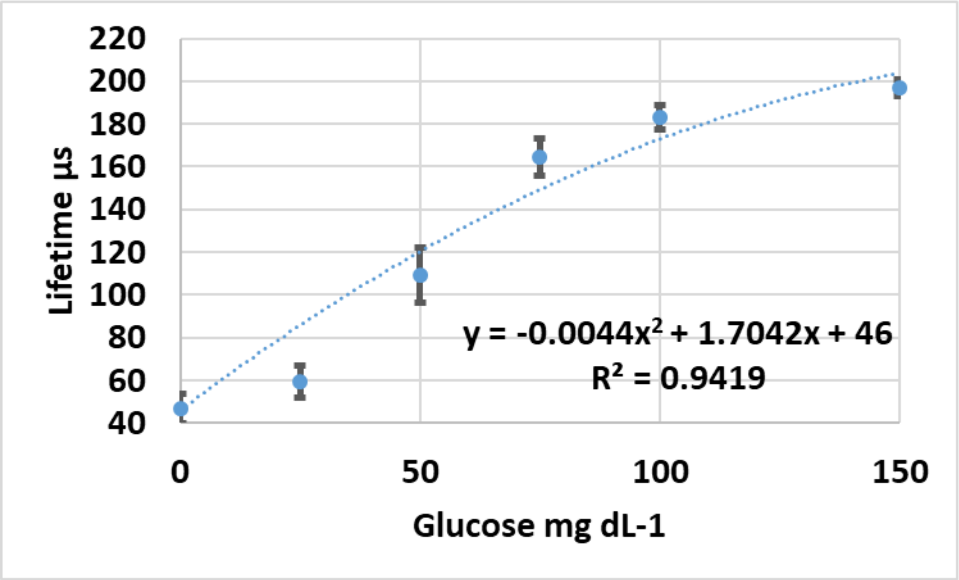

Figure S6. The second-degree polynomial fit used for the glucose sensor in 0-150 mg dL^{-1} glucose concentration.

Table S1. Comparison between polystyrene and ethyl cellulose matrix for nanoparticles.

Surfactant	Polystyrene ($\tau_{21\%}$ (μs)/ $\tau_{0\%}$ (μs)/ K_{sv} (μM^{-1}))	Ethyl cellulose ($\tau_{21\%}$ (μs)/ $\tau_{0\%}$ (μs)/ K_{sv} (μM^{-1}))
**Sodium dodecyl sulfate (SDS)**	35.2±0.8/ 306.5±0.8/ 0.034	23.2±0.6/ 244.0±3.5/ 0.041
**Cetyltrimethylammonium bromide (CTAB)**	37.2±1.8/ 273.4±6.6/ 0.029	17.3±0.2/ 224.6±11.6/ 0.052
**Pluronic F68 (PF 68)**	35.8±0.6/ 305.8±31.9/ 0.033	20.9±0.1/ 221.0±5.8/ 0.047
**Poly(dimethylsiloxane-b-ethylene oxide) (PDMS-PEG)**	33.7±0.4/ 287.8±3.7/ 0.033	20.0±0.6/ 236.3±4.1/ 0.042
